# Cerebral adrenoleukodystrophy presenting as status epilepticus: Unveiling the neurological maze

**DOI:** 10.1016/j.radcr.2024.10.018

**Published:** 2024-10-30

**Authors:** Saket Satyasham Toshniwal, S. Jiwan Kinkar, Sunil Kumar, Sourya Acharya

**Affiliations:** a Department of General Medicine, Jawaharlal Nehru Medical College, Datta Meghe Institute of Higher Education and Research, Wardha, India; b Department of Neurology, Jawaharlal Nehru Medical College, Datta Meghe Institute of Higher Education and Research, Wardha, India

**Keywords:** White matter disease, Childhood cerebral adrenoleukodystrophy, X-linked disease, Very long chain fatty acid accumulation, Childhood

## Abstract

We describe the case of a 7-year-old boy who had repeated episodes of prolonged seizures without recovery of consciousness when he arrived at a rural tertiary care teaching institute hospital in Wardha, India. Detailed history of the patient revealed that the child's symptoms began with left exotropia and visual acuity changes, progressing over 6 months to cognitive decline, hearing impairment, pseudobulbar affect, and motor issues, eventually leading to school dropout. Social isolation and difficulty walking also developed as the disease advanced. MRI brain revealed diffuse white matter lesions bilaterally with raised serum ACTH levels of 5 times the normal range associated with raised levels of tetracosanoic acid (C24) and hexacosanoic acid (C26), along with elevated C24/C22 and C26/C22 ratios. The patient was provisionally diagnosed as X linked cerebral adrenoleukodystrophy. Post treatment and stabilization, the patient was seizure-free on antiepileptic medications, however, patient developed blindness, lost mobility, became bedridden, and progressed to a vegetative state within 6 months. Adrenoleukodystrophy (ALD) is a rare X-linked genetic disorder that primarily affects men. It is caused by mutations in the ABCD 1 gene and is characterized by an abnormal build-up of very long-chain fatty acids (VLCFA) in various body tissues, which affect the spinal cord, white matter, and adrenal glands, causing progressive damage and dysfunction at each location. This case highlights the importance of early diagnosis and intervention to slow down disease progression in order to improve outcome. Also, increased awareness among healthcare professionals to help early detect the signs of this disease is of great importance.

## Introduction

ALD typically manifests between the ages of 4-10 years as in our case and the clinical presentation of ALD can vary with different phenotypes ranging from childhood cerebral ALD (CCALD) to adult-onset adrenomyeloneuropathy(AMN) and other milder forms of which CCALD which comprises of only 1 in 25000 boys worldwide making it the rarest form of ALD [1]. Adrenoleukodystrophy of adult onset is extremely uncommon, accounting for only 2%-5% of all documented cases, in contrast to its prevalence in childhood. The affected gene (ABCD1) codes for the protein involved in the metabolic pathway of very long chain fatty acids (VLCFAs) in cellular peroxisome [1]. VLCFA accumulation results in adrenal insufficiency in the adrenal cortex and demyelination in the central nervous system (CNS) [2]. About 1 in 20,000 hemizygotes and 1 in 16,800 heterozygotes are born with X-ALD.

Given the significant overlap between the clinical signs and symptoms of more frequent demyelination, cerebrovascular, or metabolic illnesses, it can be difficult to diagnose and place a patient in the right pipeline for therapy in the absence of established standards [3].

Early identification of CALD is crucial due to the potential for rapid deterioration if left untreated.

## Case presentation

A male child in his middle childhood, first kid, well-managed pregnancy, nonconsanguineous parents, with non significant family history, previously healthy, presented with recurrent episodes of prolonged seizures of generalized tonic clonic type, without recovery of consciousness. Emergency treatment was given to the patient, his airway was protected and the patient was taken on mechanical ventilator. Intravenous lorazepam 4 mg was administered and was repeated. Intravenous phenytoin 20 mg/kg was administered at a rate of 50 mg/minute, while the patient was monitored for hypotension and arrhythmia in the intensive care unit set up. The seizure activity was controlled and the patient's metabolic panel and hemodynamic status along with continuous electroencephalogram was thoroughly monitored and was kept on intravenous antiepileptic medications round the clock. The patient was eventually weaned off the ventilator and started on supportive and rehabilitative treatment measures.

Meanwhile, on detailed history taking, his parents revealed that his symptoms began with a change in visual acuity related to left exotropia and quickly progressed 6 months ago. In the next 2 months, he started having problems in school because of his lack of concentration, inability to follow directions, and the loss of basic maths problem solving skills and reading skills. In the next 4 months, the teachers noticed that the child had hearing impairment, the need to repeat instructions in order to complete activities, pseudobulbar affect, social isolation and a lack of interest in social interactions. The child dropped out of school at 6 months of disease progression since there was evidence of frequent falls and the need for assistance when walking in unfamiliar locations owing to increased visual impairment.

Differential diagnoses were taken into consideration in light of this abrupt developmental regression history such as X linked adrenoleukodystrophy, mitochondrial encephalopathies, Huntington's disease, Krabbe disease, multiple sclerosis.

**Fig. 1:**
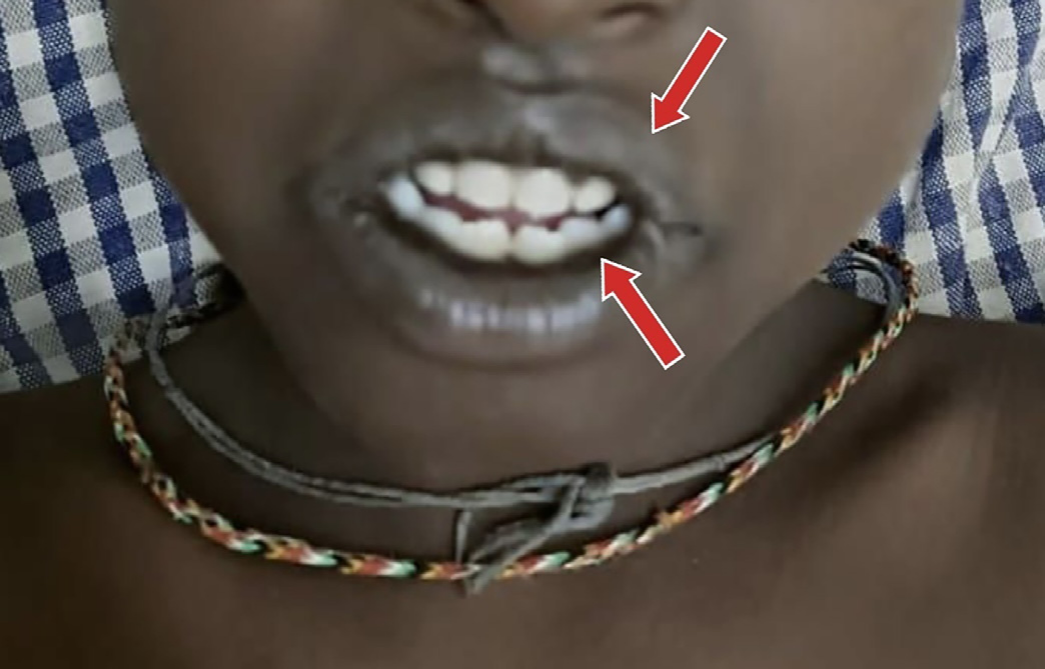
Hyperpigmentation in perioral region and gums shown with red arrows.

**Fig. 2:**
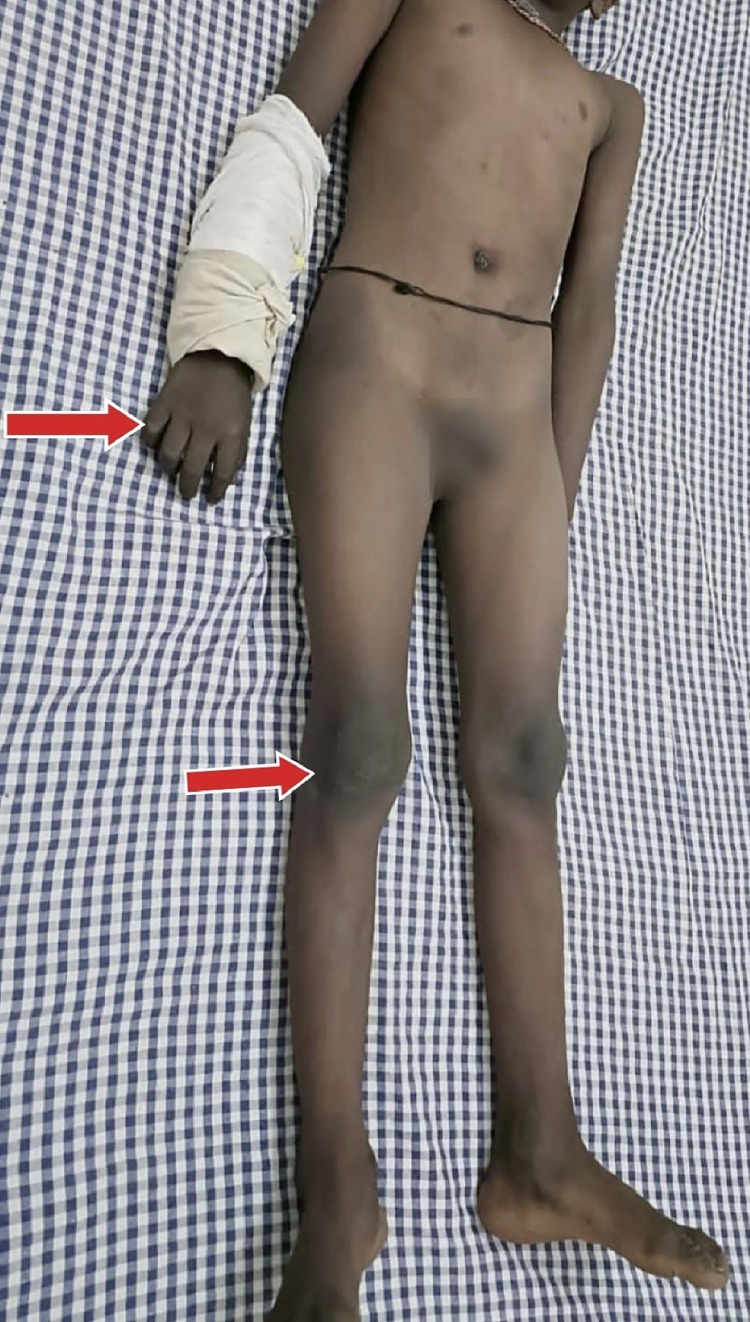
Diffuse hyperpigmentation particularly involving joints and hands shown with red arrows.

General examination of the child patient revealed diffuse hyperpigmentation of skin especially over joints, perioral area, gums and hand knuckles was a startling discovery that raised the possibility of X-ALD as shown in Fig. 1, Fig. 2.

His neurological examination revealed that he was alert, had hypoprosexia, trouble reading and calculating, had trouble with abstraction and analogy thinking for his age, had bilateral visual acuity of 20/150, had a positive Rinne test, with no changes to other cranial nerves, no changes to strength or sensitivity, but with spasticity in all 4 limbs with exaggerated deep tendon reflexes in all 4 limbs with bilateral extensor plantar response, walked with a spastic gait, and did not exhibit ataxia.

**Fig. 3:**
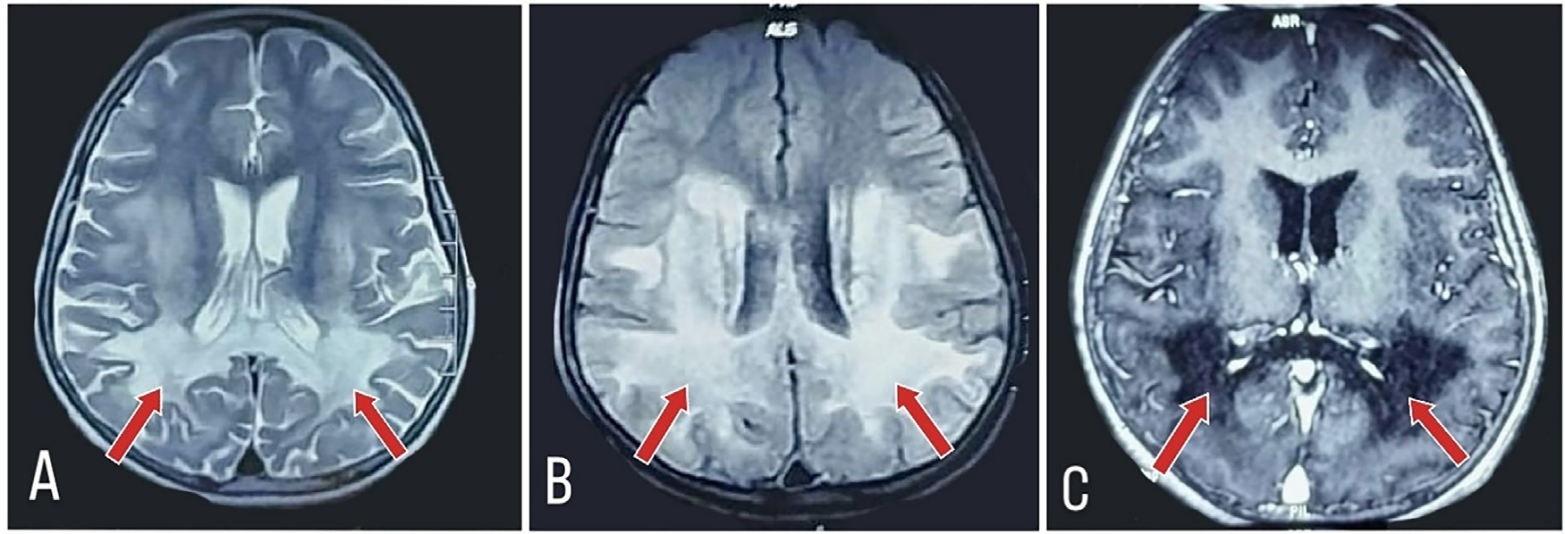
(A) MRI brain T2 weighted axial sections showing bilateral parieto-occipital white matter abnormal signals in periventricular and subcortical planes marked with red arrows. (B) MRI brain (FLAIR) axial sections showing hyperintensities in bilateral parieto-occipital regions with white matter abnormal signals in periventricular and subcortical planes marked with red arrows. (C) MRI brain showing no significant enhancement on post contrast scan marked with red arrows.

MRI brain with contrast was done to rule out any structural defects leading to status epilepticus as shown and explained in Fig. 3.

On further investigations, owing to the possibility of X- ALD, patient had 5 times the normal amount of ACTH, indicating adrenal insufficiency, which at the time was asymptomatic. Supplementing with hydrocortisone was initiated. A VLCFA profile was consistent with X-linked adrenoleukodystrophy and showed increased levels of tetracosanoic acid (C24) and hexacosanoic acid (C26), along with elevated C24/C22 and C26/C22 ratios, and an altered discriminant function between men and women with peroxisomal disease. Raised levels of serum triglyceride levels were found on fasting lipid profiles. Arterial blood gas analysis was done and was within normal limits with normal serum lactate levels ruling out lactic acidosis, hence ruling out mitochondrial metabolic disorders. Serum glycosphingolipid markers were done and found to be negative ruling out Krabbe's disease. His childhood presentation showed a fast progression in his clinical picture. Following diagnosis confirmation, the MR images were assessed using the Loes score to determine the extent of neuroanatomical involvement, with a final score of 22 points out of the maximum score of 34 points. Each neuroanatomical area has been assigned a maximum score of 4 depending on the sites involved and some neuroanatomical areas with a maximum score of 2 whereas each region is given a score of 0 for normal, 0.5 for unilateral involvement, and 1 for bilateral involvement or atrophy. The maximum score is 34. The final breakdown of the MRI severity score i.e., Loes score for our patient is shown in Table 1. MRI plays a crucial role in diagnosis, assessing severity, prognosis and monitoring of lesions, hence plays a pivotal role in CALD.

**Table 1:** Loes score breakdown of the patient.

Sr. No	Neuroanatomy	Patient score	Total score
1	Parietal Occipital White Matter	2	4
2	Anterior Temporal White Matter	3	4
3	Frontal White Matter	2	4
4	Corpus Callosum	2	4
5	Visual Pathway	3	4
6	Auditory Pathway	4	4
7	Pyramidal System	1	2
8	Cerebellum	1	2
9	Basal Ganglia	1	1
10	Anterior Thalamus	1	1
11	Global Atrophy	2 (Moderate)	3 (Severe) + 1 (Brainstem)
	Total Score	22	34

The interpretation of Loes score is shown in Table 2. Our patient had a Loes score of 22, hence falls in the moderately severe abnormalities category. Bone marrow transplant was not indicated in this case because the benefits outweigh the potential risks as the Loes scale depicted a score of more than 9. Instead, supportive management, genetic counselling, and interdisciplinary follow-up were started. Genetic studies were also advised, but could not get it done due to financial constraint of the family of the patient.

**Table 2:** Interpretation of Loes score.

Sr. No.	Loes Score	Interpretation
1	0	Normal
2	1-9	Minimal abnormalities
3	10-14	Mild abnormalities
4	15-19	Moderate abnormalities
5	20-24	Moderately severe abnormalities
6	25-29	Severe abnormalities
7	30-34	Very severe abnormalities

On subsequent follow up, the patient was seizure free on antiepileptic medications of tablet levetiracetam 500 mg twice a day, with tablet phenytoin at 8mg/kg/day with 2 divided doses. However, the patient developed blindness and lost his capacity to walk on his own after 3 months and got bedridden. The patient deteriorated neurologically within the next 6 months which led to an apparent vegetative state.

## Discussion

X-ALD is the most prevalent peroxisomal disorder worldwide characterized by demyelination of the nervous system, accumulation of long-chain fatty acids and adrenal insufficiency which occurs rarely in the pediatric population with an estimated frequency of 1 in 17,000 to 35,000 live births [4]. Clinical manifestations include ataxia, blindness, hearing loss, behavioral issues, epileptic convulsions that progress to psychosis, mental decline, and ultimately death. There have only been a few documented cases of ALD that manifested as catastrophic status epilepticus [4]. Prior to neurological symptoms, abnormal skin pigmentation and other signs of adrenal insufficiency may be noticeable. Adrenal symptoms may not even manifest. The diagnosis of X-ALD is confirmed by analyzing the plasma levels of VLCFAs and identifying aberrant mutations in the ABCD1 gene [5,6]. Magnetic resonance imaging (MRI) of brain is an essential diagnostic and surveillance modality for paediatric X-linked ALD, according to Kim et al.'s study. It helps distinguish ALD from other disorders and allows for early detection [7]. Nascimento et al. reported a case with a 2-year old who showed hyperpigmentation and fatigue [8]. MRI findings in the case described by Nascimento et al, revealed bilateral symmetrical hyperintense white matter lesions in parieto-occipital region along with involvement of posterior limb of internal capsule, like in our case where we observed bilateral involvement of parieto-occipital regions [8]. The prognosis was done using MRI. Moser HW assessed the degree of brain MRI abnormalities in 372 individuals using the MRI-scoring method developed by Loes et al. [8,9].

Rai PS, et al described 2 cases of CALD; whose initial symptoms included behavioral changes, irritability, and academic decline with development of cognitive impairments, motor dysfunction, and seizures, leading to significant neurological deterioration depicting typical clinical trajectory of the classic form in these cases [10]. MRI findings in the cases described by Rani PE, et al included classic CCALD pattern hyperintensities in bilateral parieto-occipital region in 1 case while the other case showed atypical involvement of frontal white matter along with corticospinal tract and brainstem, unlike our case where the lesions were observed in bilateral parieto-occipital region on MRI brain [10].

MR spectroscopy seems to be a practical, noninvasive method for monitoring ALD patients [11,12].

MRI places a crucial role in diagnosis of CCALD via early detection of lesions, monitoring disease progression, differentiating from other demyelinating disorders by assessing the pattern and location of lesions and also assess severity of the disease by Loes score [11]. MRI also helps in assessing the response to treatment by monitoring the stabilization and progression of brain lesions [12].

Polgreen LE, et al in their study found that patients with a delayed diagnosis of X-ALD exhibited more limited functionality and substantial brain impairment at the time evaluation for hematopoietic cell transplantation (HCT) examination and were 3.9 times more likely to die than boys who received an early diagnosis of X-ALD [13].

In the US, state-mandated new-born screening requires the use of the Recommended Uniform Screening Panel, which includes X-ALD [14]. A reliable diagnosis requires carrier gene sequencing and genetic testing for the mutant ABCD1 gene in males [15]. However, the adult subgroup does not contain many individuals of this type. This case highlights the significance of differentiating adult-onset X-linked adrenoleukodystrophy from other neurodegenerative diseases that are common in this age group.

## Conclusion

By documenting a detailed case of a 7-year-old boy, this report highlights the clinical features and progression of CALD, aiding healthcare professionals in recognizing the disease at an earlier stage. Symptomatic and supportive care, including physical therapy and special education services to spread awareness about the disease especially in the rural setup can help improve quality of life for the affected individual as well as the family of the sufferer as this disease can have a significant mental and social impact on the affected child and their family.

This case report also serves as an educational tool for medical professionals, researchers, and students, emphasizing the need for heightened awareness of rare genetic disorders and the importance of comprehensive evaluation in cases of unexplained neurodegenerative symptoms.

## Patient consent

A written and informed consent was taken from the caretaker (father) of the child.

## Disclosure

The authors hereby certify that the work shown here is genuine, original and not submitted anywhere, either in part or full.
